# Van Neck-Odelberg Disease: A Systematic Review of Literature and Case Presentation

**DOI:** 10.7759/cureus.72751

**Published:** 2024-10-31

**Authors:** Muhammad Arham Sahu, Abdullah Tahir, Muhammad Ashal Sahu, Tahir Rafiq

**Affiliations:** 1 Trauma and Orthopaedics, Hereford County Hospital, Wye Valley NHS Trust, Hereford, GBR; 2 Medicine, School of Medical Sciences, University of Manchester, Manchester, GBR

**Keywords:** groin pain, ischiopubic osteochondritis, paediatric groin pain, paediatric orthopedics, van neck-odelberg disease

## Abstract

Van Neck-Odelberg disease (VND) is a term used to describe ischiopubic osteochondritis, which typically presents with atraumatic unilateral groin pain. We performed a systematic review of the existing literature on reported cases of VND to collate what is already known. We also present a new case of VND to add to the relatively small body of literature on the topic.

A systematic literature review was performed in July 2024 of PubMed, Medline, and Cochrane databases using the MeSH terms “van Neck Odelberg” to identify published articles. Inclusion criterion was defined as articles that provide individual details on cases of VND. Literature reviews without individual case details were excluded. Two authors independently screened titles and abstracts according to relevance and content. A total of 16 case reports and four case series were included in the final review, excluding our own case presentation.

This review included 43 cases of VND (28 males and 15 females). There was no significant difference in average age of presentation between males and females. Majority of VND cases presented with unilateral pain (n=37), mainly in the groin (n=25) and typically non-radiating (n=18). Pain associated with VND most commonly occurred on the side of the non-dominant lower limb (n=8).

Review findings demonstrated a common pattern of features associated with the presentation of VND, which included unilateral non-radiating groin pain occurring on the side of the non-dominant lower limb.

Our case of a nine-year-old, left-foot dominant, boy who presented with atraumatic right-sided groin pain was in keeping with others reported from our review. MRI confirmed the diagnosis of VND in our case; repeat MRI after a period of activity modification and use of non-steroidal analgesics showed improvement in radiological appearance, and the patient showed improvement in function.

## Introduction

Ischiopubic synchondrosis is a type of synchondrosis joint, which typically exists during skeletal development and is temporary until its final fusion during puberty [1-3]. The ischiopubic synchondrosis is present bilaterally, joining the ischium with the pubic ramus, and first begins to develop in the fifth and sixth months in utero. It is a cartilaginous strip that is located between the junction of two ossification centres, the pubic centre located superomedially and the ischial centre located inferolaterally [3,4]. Development and subsequent ossification of this joint is a normal physiological process that is usually asymptomatic in the majority of cases; however, this is not always true [5].

In 1924, van Neck and Odelberg described radiological changes observed in the ischiopubic region in infants and grouped these changes as osteochondrosis ischiopubica [6,7]. In cases of ischiopubic osteochondritis, or alternatively van Neck-Odelberg disease (VND), atraumatic unilateral pain localising to the ischiopubic region of the affected hip and subsequent antalgic gait are the main symptoms [8,9]. We conducted a systematic review of the existing literature on VND and summarised the pertinent learning points, in addition to presenting our own case of VND.

## Case presentation

A nine-year-old, left lower limb dominant, boy presented with his parents to the emergency department with a three-week history of antalgic gait and complaint of pain localising to the right-sided groin and radiating to the ipsilateral upper thigh. The patient and their parents denied any history of trauma, fever, weight loss or night sweats. They also denied any pain in the knee. They described the pain as a dull ache that intensified upon weight bearing and mobilising, but would be relieved with rest.

Upon examination, there were no overlying skin changes or swellings over the affected hip. Tenderness was elicited on palpation over the superior anterolateral aspect of the thigh, around the greater trochanter and over the hamstrings. There was minimal joint tenderness on palpation, with a full range of hip movement and full internal and external rotation. At extremes of hip flexion, the patient complained of pain in the hamstrings. Examination of the knees did not yield anything abnormal, and the patient also had a normal spinal examination.

**Figure 1:**
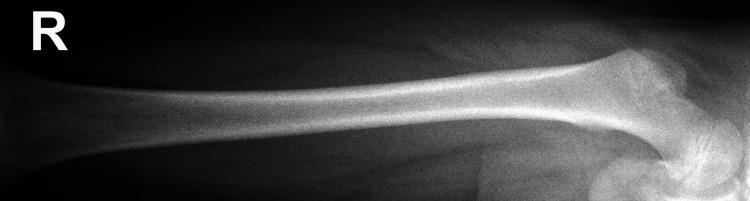
X-ray (anterior-posterior view) of the right femur. No bony abnormality, acute injury, or lytic or destructive bony lesion is seen. Right hip joint appears normal.

**Figure 2:**
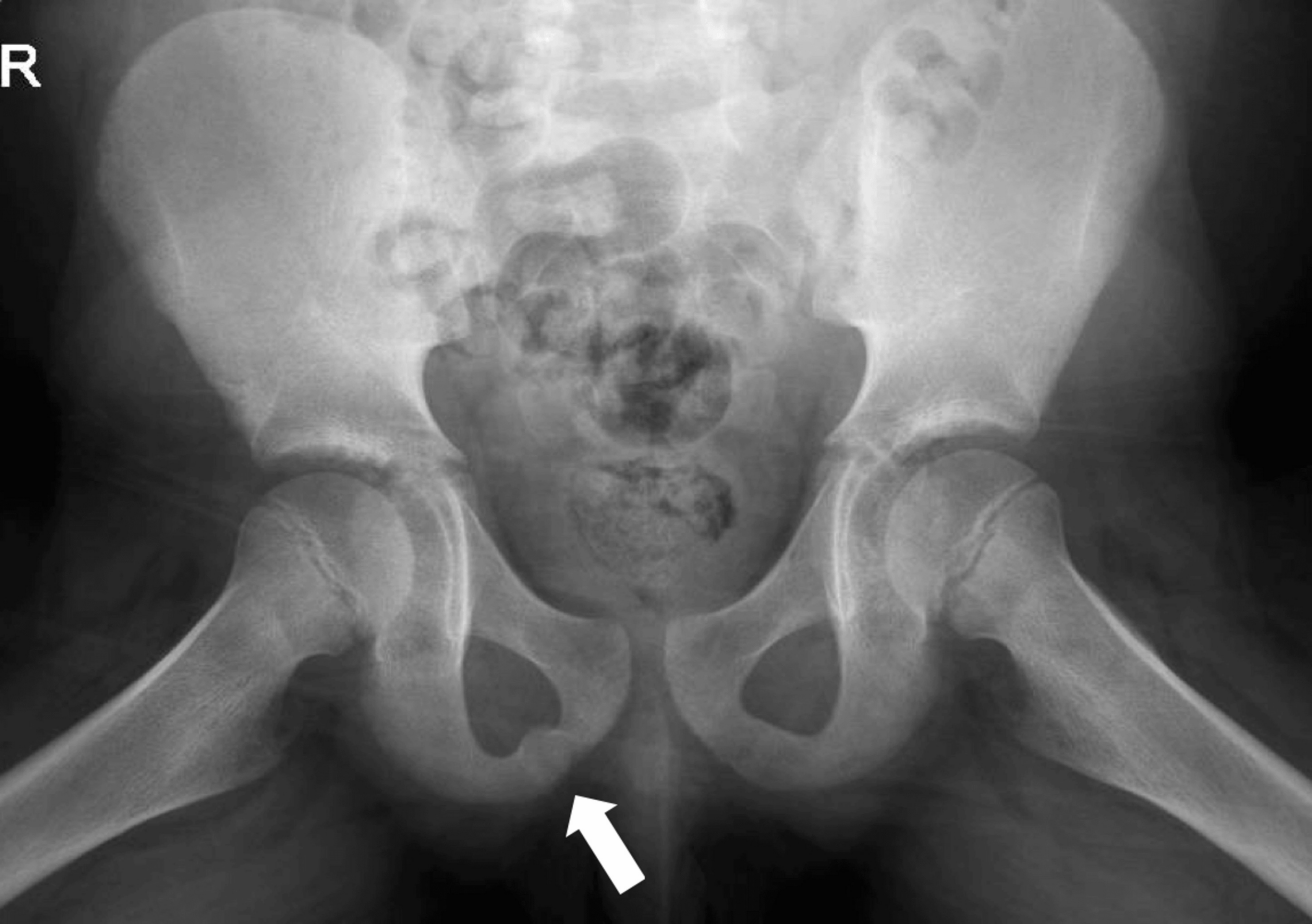
X-ray (anterior-posterior radiograph view) of the pelvis. Right-sided inferior pubic ramus shows focal bubbly lucency and enlargement, which is not visible on the included left-sided inferior pubic ramus. No periosteal reaction is visible along the right-sided inferior pubic ramus. White arrow points to the round focal enlargement on the right-sided inferior pubic ramus, suggestive of van Neck-Odelberg disease.

Radiographs were taken of the patient’s hip and femur to exclude any long-bone pathology that may explain the patient’s symptoms (Figures 1, 2). X-ray revealed a focal bubbly lucency and enlargement on the right-sided pubic ramus, which was not present on the contralateral side (Figure 2).

**Figure 3:**
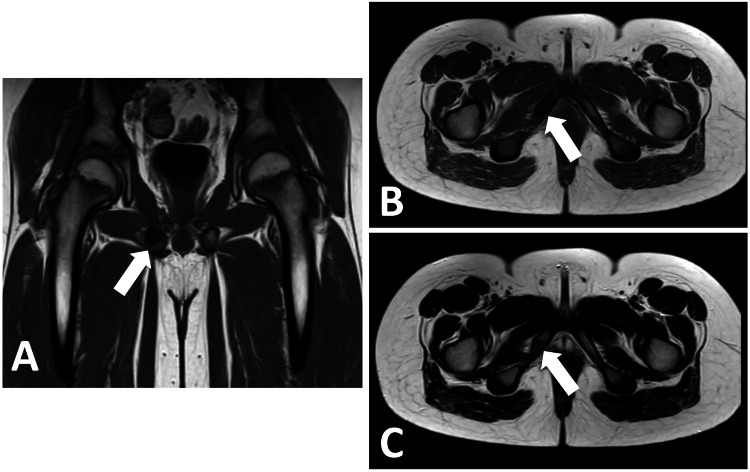
Original MRI scan at presentation following X-ray. (A) MRI T1-weighted sequence (coronal view). (B) MRI T1-weighted sequence (axial view). (C) MRI T2-weighted sequence (axial view). MRI examination confirms asymmetric bilateral inferior pubic ramus with focal enlargement with cortical break and marked surrounding oedema extending into right pubic body as well as right ischial tuberosity. Minimal periosteal soft tissue oedema is also noted along the right inferior pubic ramus, although there is no hyperintensity in the adductor tendons or surrounding muscles. Left inferior pubic ramus appears normal. Both hip joints and femur appear normal. Visible pelvic and thigh musculature appears normal. No definite soft tissue mass is seen in the muscle compartments. No avulsion injury is seen, and tendons in the pelvis appear normal. MRI findings confirm van Neck-Odelberg disease. White arrows in each MRI sequence point to the focal enlargement at the right inferior pubic ramus and surrounding oedema, which confirm van Neck-Odelberg disease.

Based on the atraumatic history, examination findings and the radiological findings on X-ray, the patient underwent an urgent MRI scan to exclude more sinister pathology. As shown in Figure 3, MRI confirmed radiological X-ray findings of an asymmetrical right-sided inferior pubic ramus with focal enlargement cortical break and surrounding oedema extending into the right-sided pubic body and the right-sided ischial tuberosity. There was no evidence of soft tissue masses, muscular abnormality or avulsion injury.

Taking together the clinical information with the imaging findings, a diagnosis of VND was made. The mainstay of management for VND includes non-steroidal analgesic drugs (NSAIDs) and activity modification: the patient was asked to avoid participation in high-impact sports and related activities, which are likely to place stress on the joints and consequently worsen the degree of inflammation of the ischiopubic synchondrosis, resulting in more pain.

**Figure 4:**
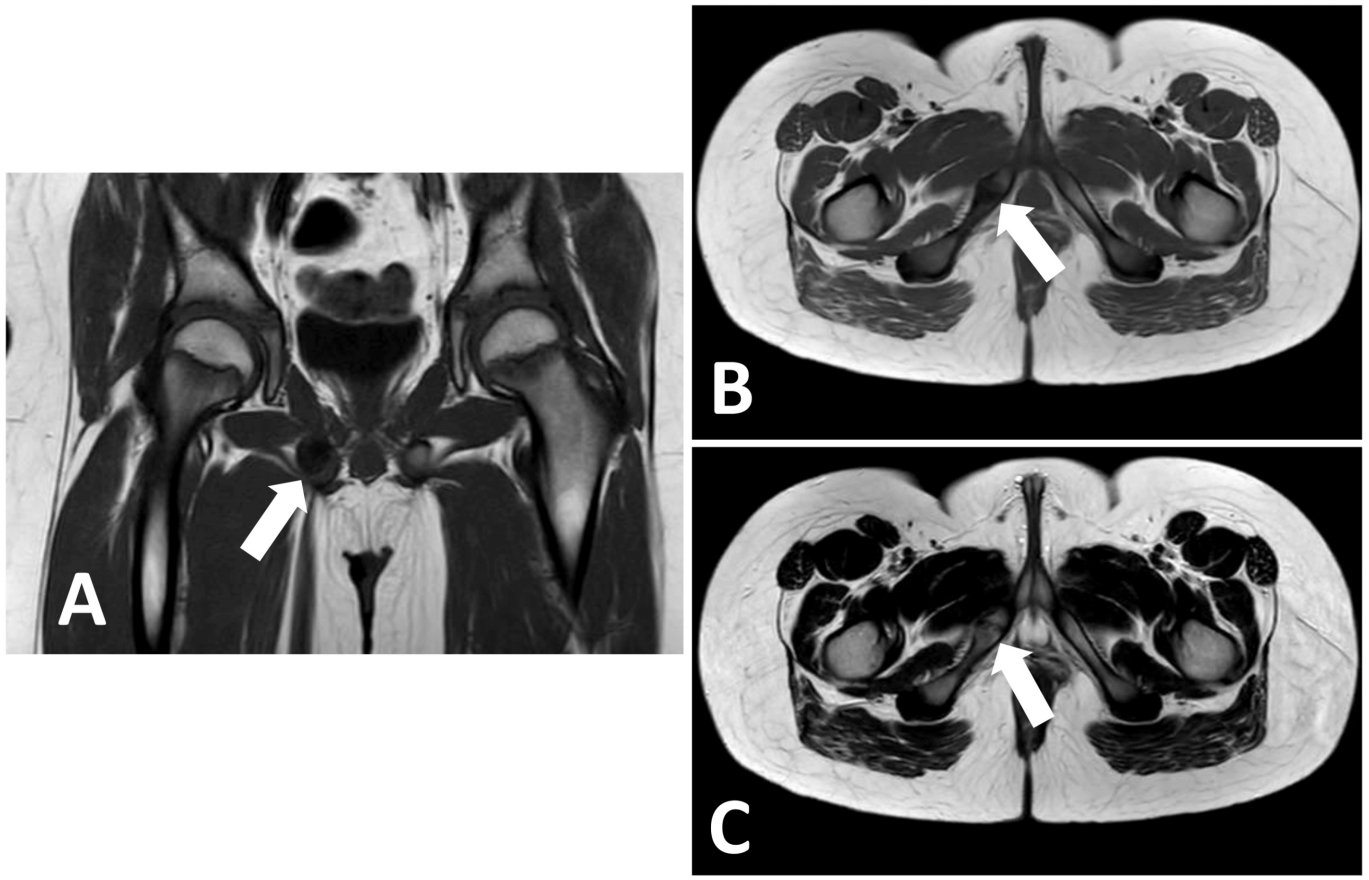
Repeat MRI scan. (A) MRI T1-weighted sequence (coronal view). (B) MRI T1-weighted sequence (axial view). (C) MRI T2-weighted sequence (axial view). Repeat MRI examination shows significantly reduced marrow oedema in the right inferior pubic ramus and also regressed oedema in the right pubic body and right ischial tuberosity, as well as resolution of periosteal soft tissue oedema. Residual oedema in focally enlarged right inferior pubic ramus, with cortical break noted. Left-sided pubic rami are normal. Both hip joints are normal. No other marrow oedema is seen in the pelvic bones. Compared to the previous MRI scan, there is good imaging improvement with considerably reduced bony marrow oedema and surrounding periosteal soft tissue hyperintensity in the right ischiopubic synchondrosis, although focally enlarged right inferior pubic ramus with cortical break remained the same. White arrows in each MRI sequence point to the focal enlargement in keeping with VND that has reduced in size at the right inferior pubic ramus, with improved surrounding oedema.

The patient was planned for a repeat MRI scan in eight weeks’ time (Figure 4), which demonstrated overall improvement from the previous scan findings; there was significantly reduced bone oedema and resolving oedema around the right-sided ischial tuberosity and right-sided pubic body. The patient will continue to receive NSAIDs and continue activity modification with regular follow-up and repeat imaging at regular intervals to monitor recovery. The patient is expected to make a full recovery with no complications and return to their normal physiological baseline once skeletal maturation is achieved.

## Discussion

Systematic review

Systematic Review Methods

A systematic review of literature was performed in July 2024 of PubMed, MedLine, and Cochrane databases using the MeSH terms “van Neck Odelberg” and “ischiopubic osteochondritis” to identify published articles. The search was restricted to articles published in English. Inclusion criteria was defined as articles that included cases of VND and mentioned information on presenting features, diagnostic methods and management plans employed. Articles which did not include case details, such as narrative reviews, biomechanical and imaging studies, were excluded. Title and abstract screening was performed by two authors independent of each other to ensure inclusion of relevant case reports and series. A total of 20 articles, 16 case reports and four case series were included in the final review, excluding our own case presentation.

Systematic Review Results

A total of 43 cases were included in this systematic literature review (28 males and 15 females) (Table 1). From the literature, we found that the average age of presentation is approximately 10 years, with no significant difference between males and females. The majority of cases of VND presented with unilateral pain (n=37) rather than bilateral pain (n=6); the pain was found mainly in the groin pain (n=25), whilst some presented primarily with either gluteal pain (n=5) or hip pain (n=5). A small minority presented with primary thigh pain (n=2). Patients were typically found to have non-radiating pain in most cases (n=18), but a small number presented with radiating pain to either the knee (n=1) or the anterior thigh (n=3). Of the cases in which lower limb dominance was mentioned, pain most commonly occurred on the non-dominant side (n=8).

**Table 1:** Details of included articles in the systematic review *Range of ages given in the included cases of this article.

Year of Publication	Author	Article Type	Number of Cases	Age of Cases (Years)	Male:Female	Area of Pain
2010	Oliveira [2]	Case report	1	8	Male	Groin
2016	Beyitler and [10]	Case report	1	7	Male	Groin
2017	Chaudhari et al. [11]	Case report	1	12	Male	Groin
2017	Mixa et al. [12]	Case report	1	15	Female	Groin
2017	Morante et al. [13]	Case report	1	8	Male	Hip
2018	Pirimoglu and Sade [14]	Case report	1	8	Male	Groin
2020	Ceri and Sperati [15]	Case report	1	8	Male	Groin
2020	Walter et al. [16]	Case report	1	10	Male	Groin
2021	Macarini et al. [9]	Case series	2	8; 12	Male; Male	Groin; Hip
2021	Narayanan et al. [17]	Case report	1	11	Male	Gluteal
2021	Sabir et al. [18]	Case report	1	14	Male	Gluteal
2021	Schneider et al. [19]	Case series	21	8-13*	13 males, 8 females	10 groin, 2 gluteal, 1 hip, 2 thigh
2021	Tam et al. [20]	Case report	1	12	Male	Groin
2022	Camacho et al. [5]	Case report	1	15	Male	Groin
2022	Fonseca et al. [21]	Case report	1	6	Male	Groin
2022	Korkmazer et al. [22]	Case series	2	10; 7	Female; female	Groin, groin
2023	Hursoy et al. [23]	Case series	2	8; 4	Female; male	Hip, groin
2024	Afaque et al. [24]	Case report	1	13	Female	Groin
2024	McLoughlin et al. [25]	Case report	1	9	Female	Hip
2024	Wang et al. [26]	Case report	1	88	Female	Gluteal

Systematic Review Findings

VND, whilst carrying the description of a “disease”, has been shown in the majority of cases published in the literature to be self-limiting and without the need for intervention other than simple analgesia and activity modification [8,12]. The case we have presented in this article follows the same trend. With a growing body of evidence on the progression of VND through previously published cases, it is sensible to consider VND to be a physiological variant of normal developmental anatomy rather than a pathological process requiring invasive intervention [19].

VND has been associated with foot dominance, with the majority of published cases demonstrating occurrence on the non-dominant side. This trend could be explained by greater cumulative strain placed on the non-dominant lower limb hamstrings and adductor magnus muscles, all of which originate from the ischial tuberosity [1,27]. The non-dominant foot would not enter the swinging phase as often in comparison to the dominant foot, the non-dominant leg would typically spend a greater time weight-bearing; the increased stress from this would result in increased tension on the hamstrings and, in turn, pull on the ischial tuberosity. The increased mechanical stressors placed on the ischial tuberosity may lead to insult to the ischiopubic synchondrosis, resulting in subsequent inflammation and symptoms observed in cases of VND [1,27]. This, in turn, would fit with the clinical presentation of typically unilateral groin pain, as has been found in the majority of reported cases of VND. Our case also demonstrates a similar trend towards VND occurring on the non-dominant side and presenting with unilateral groin pain. However, there does remains a paucity of high-quality evidence in previously published literature to significantly support this theory.

The rarity of VND in the general population poses a significant risk for misdiagnosis; some important differentials for VND include ischiopubic osteochondritis and primary bone tumours [12,27]. Previously reported cases of VND demonstrate how, on X-rays and CT scans, it can appear as a lesion highly suspicious of a tumour [28]. Differentiating VND from tumour will require a combination of clinical history and examination, laboratory investigations and diagnostic imaging. From reviewed case reports, and indeed from our own experience from the case presented above, MRI remains one of the most useful imaging modalities for the diagnosis of VND and exclusion of other important differentials that may present with similar clinical features and history [1,12].

## Conclusions

VND is an important, albeit rare, variant of the process of physiological skeletal maturation of the ischiopubic synchondrosis. Given the typical features of its presenting history, namely unilateral pain, most commonly in the groin, in the absence of any trauma in an otherwise healthy patient, MRI proves to be the most useful imaging modality for excluding more sinister pathologies and diagnosing VND.

Our systematic review of existing published literature has shown that VND generally follows a common presentation of non-dominant lower limb unilateral groin pain. The case we present is largely in keeping with these trends observed and adds to the growing body of literature surrounding VND. Through increasing awareness and knowledge of this variant, patients and their families will be spared needless invasive investigation and related anxiety, whilst being provided with reassurance of the self-limiting nature of VND.
